# A phase-transited lysozyme coating doped with strontium on titanium surface for bone repairing via enhanced osteogenesis and immunomodulatory

**DOI:** 10.3389/fcell.2024.1506671

**Published:** 2025-01-06

**Authors:** Yu Zhang, Yu Chen, Yidan Shi, Hongkun Hu, Zhongyu Dai, Zhichen Liu, Xuanan Li

**Affiliations:** ^1^ Hunan Cancer Hospital, The Affiliated Cancer Hospital of Xiangya School of Medicine, Central South University, Changsha, China; ^2^ The High School Attached to Hunan Normal University, Changsha, China; ^3^ Department of Orthopedic Surgery, National Clinical Research Center for Geriatric Disorders, Xiangya Hospital, Central South University, Changsha, China

**Keywords:** strontium, titanium, lysozyme, immunomodulation, osteogenesis

## Abstract

**Introduction:**

Titanium is currently recognized as an excellent orthopedic implant material, but it often leads to poor osseointegration of the implant, and is prone to aseptic loosening leading to implant failure. Therefore, biofunctionalization of titanium surfaces is needed to enhance their osseointegration and immunomodulation properties to reduce the risk of implant loosening. We concluded that the utilization of PTL-Sr is a direct and effective method for the fabrication of multifunctional implants.

**Methods:**

In this Study, phase-transited lysozyme (PTL) is deposited onto the surface of titanium (Ti) to construct a functional coating and strontium chloride solution was utilized to produce PTL coatings with Sr^2+^. The characterization of the strontium-doped PTL coatings (PTL-Sr) was tested by scanning electron microscopy (SEM), energy dispersive X-ray spectroscopy (EDX) and inductively coupled plasma atomic emission spectroscopy (ICP-AES). A series of cell and animal experiments were conducted to investigate the biological functions of PTL-Sr coatings.

**Results:**

The characterization indicates the successful preparation of PTL-Sr coatings. *In vitro* cellular experiments have demonstrated that it promotes M2 macrophage polarization and reduces inflammatory mediator production while promoting osteogenic differentiation of bone merrow mesenchymal stem cells (BMSCs). The *in vivo* subcutaneous implantation model demonstrated its good immunomodulatory and angiogenic properties.

**Discussion:**

Titanium with PTL-Sr coatings promote biomineralization and immunomodulation, which is suitable for orthopedic applications. Further mechanistic exploration and studies using animal models is necessary to enhance the understanding of the clinical applicability of modified titanium.

## 1 Introduction

Autologous bone grafting is considered the gold standard for bone defect repair (Van Heest and Swiontkowski, 1999). However, the scarcity of autologous bone sources, coupled with complications such as infection and poor healing, limits its application (Van Heest and Swiontkowski, 1999; Kawecki et al., 2018). Designing orthopedic implants with excellent performance to replace autologous bone grafts for bone defect repair has become a current research hotspot.

Titanium (Ti) is recognized as an exceptional orthopedic implant material due to its favorable biocompatibility, ease of processing, and radiation resistance (Ma et al., 2014; Jiang et al., 2023). However, the surface inertness of Ti implants is detrimental to the effective integration of the implant with bone, increasing the likelihood of aseptic loosening and inadequate bone bonding (Zhu et al., 2016; Griffiths et al., 2021; Wang et al., 2023). The lack of necessary bioactivity makes Ti implants insufficient in promoting osteogenesis, which increasing the likelihood of implant failure (Bai et al., 2021). Mechanically adhering Ti implants to bone defect areas does not fully initiate the biological cascade necessary for tissue repair Therefore, endowing Ti implants with functional characteristics through surface modification or doping has significant scientific and clinical application potential.

Implants are perceived as foreign bodies by host tissues (Trindade et al., 2016), and focusing solely on the osteogenic and angiogenic properties of implants while neglecting the inflammatory responses they induce is inadequate. Increasing research attention has been paid to the role of immune cells in influencing bone remodeling and regeneration (Bai et al., 2021; Xu et al., 2021). The early inflammatory response of immune cells to biomaterial surfaces determines the final outcome of bone defect repair (Lee et al., 2019). Strontium (Sr), as an essential trace element in the human body, is associated with the regulation of bone metabolism and has pro-angiogenic properties (Li et al., 2019; Geng et al., 2021). Sr^2+^ can induce the differentiation of osteoblastic precursors and stimulate osteoblasts to produce new bone matrix. Various biomaterials doped with Sr^2+^ have shown enhanced osteogenic properties (Okuzu et al., 2017; Geng et al., 2021). Furthermore, extensive studies indicate that Sr^2+^ can induce the polarization of macrophages toward the M2 phenotype (Lourenço et al., 2019; Xu et al., 2021). This process helps resolve inflammation and creates a favorable local immune microenvironment, which subsequently promotes osteogenesis and tissue repair (Jin et al., 2019). M2 macrophages secrete anti-inflammatory cytokines, such as IL-4, IL-10 and transforming growth factor-β (TGF-β), which promote tissue remodeling, repair, and wound healing.

Phase-Transited Lysozyme (PTL) coating is an excellent surface modification for materials (Gao et al., 2016). Tri (2-carboxyethyl) phosphine (TCEP) can disrupt disulfide bonds within lysozyme, inducing a phase transition in aqueous solutions that results in the formation of PTL (He et al., 2023). PTL can be deposited within 2 h, and studies have indicated that PTL coatings can stably adhere to various substrates (e.g., titanium, bacterial cellulose membranes, polyether ketone), forming a particulate network structure (Gao et al., 2016; He et al., 2023). PTL coatings contain more functional groups and charges, which facilitate the adsorption of ions such as Ca^2^⁺ and Cu^2^⁺ through chemical interactions or electrostatic bonding (Yang et al., 2018; He et al., 2023). Therefore, it is hypothesized that grafting Sr^2+^ onto Ti surfaces via PTL coating is feasible.

In this study, PTL was deposited on Ti surfaces to construct a functional coating, which were subsequently grafted with Sr^2^⁺ using a strontium chloride solution. The modified titanium is expected to exhibit effective osteogenic performance and immunomodulatory properties. The biological functions of the modified titanium were evaluated through *in vitro* cell experiments and animal models. Overall, the PTL-coated and Sr^2+^-grafted modified titanium has the potential to serve as a multifunctional orthopedic implant that promotes biomineralization and immunomodulation.

## 2 Materials and methods

### 2.1 Preparation of modified titanium

Titanium plates were cut into cylindrical discs (thickness: 0.2 cm, diameters: 8 mm and 20 mm), and then polished with sandpaper (400Cw and 1500Cw). The Ti discs were subjected to ultrasonic cleaning sequentially in methanol, anhydrous ethanol, and deionized water.

Lysozyme solution (2 mg/mL) and TCEP solution (50 mmol/L, adjusted to pH 7.4 with NaOH) were prepared using HEPES buffer (10 mmol/L) as the solvent. The lysozyme solution and TCEP solution were mixed in a 1:1 ratio to produce a phase transition buffer. Within the pH range of 6-9, lysozyme could form the phase-transited protein product and effectively deposit, resulting in the formation of a PTL coating on the material surfaces (Gao et al., 2016).

Ti discs were immersed in the phase transition buffer at room temperature for 2 h. They were then washed with deionized water to remove any unbound PTL. Subsequently, Ti discs with the PTL coating were immersed in a SrCl_2_ solution (100 mM) at room temperature for 24 h. Afterward, the discs were washed with deionized water to remove any unincorporated Sr^2+^. The modified titanium was named Ti@PTL/Sr, while the Ti discs with only the PTL coating were named Ti@PTL.

All materials used for biological experiments were sterilized using ethylene oxide.

### 2.2 Characterization of modified titanium and Sr^2+^ releasing detection

The surface morphology of the modified titanium was observed using scanning electron microscopy (SEM, TESCAN, Brno, The Czech Republic). The distribution of various elements on the modified titanium surfaces was characterized using X-ray energy dispersive spectrometry (EDS, TESCAN, Brno, The Czech Republic). The static water contact angle on the surface of the modified titanium was measured using a contact angle testing system (JC 2000C, China). The chemical composition of the modified titanium was analyzed using X-ray photoelectron spectroscopy (XPS, AXIS SUPRA+, Kratos Analytical Co. Ltd., Japan).

The release of Sr^2+^ from the modified titanium was evaluated using inductively coupled plasma-optical emission spectroscopy (ICP-OES, Leeman Labs, USA). The modified titanium was immersed in 2 mL of deionized water, and 1 mL of solution was collected at specific time points (days 1, 3, 5, 7, and 10). To maintain a consistent volume for subsequent collections, 1 mL of deionized water was added after each collection.

### 2.3 Cell culture

Totally 2 types of cells were used in this study: rat bone marrow mesenchymal stem cells (BMSCs) and the mouse monocyte/macrophage cell line RAW264.7. BMSCs were cultured in α-MEM medium (NEST Biotechnology, Wuxi, China) supplemented with 10% fetal bovine serum (FBS, JYK-FBS-301, INNER MONGOLIA JIN YUAN KANG BIOTECHNOLOGY CO., LTD.) and 1% penicillin/streptomycin/amphotericin B solution (P/S/A, BioChannel Biological Technology Co. Ltd.). RAW264.7 cells were cultured in DMEM medium (BasalMedia Co., Ltd.) supplemented with 10% FBS (Umedium HeFei China) and 1% P/S/A. Osteogenic induction medium was prepared by adding dexamethasone (0.1 μM, TargetMol, United States), vitamin C (10 μM), sodium β-glycerophosphate (10 mM) to the α-MEM. All cells were cultured in a 37°C incubator with 5% CO₂.

### 2.4 Biocompatibility of modified titanium

In 48-well plates (NEST Biotechnology, Wuxi, China), BMSCs or RAW264.7 cells (5,000 cells/well) were seeded on the modified titanium surfaces. At 1 day and 3 days, the culture medium was replaced with 10% Cell Counting Kit-8 (CCK-8, Shandong Sparkjade Biotechnology Co., Ltd.) solution (200 µL/well), and co-incubated with cells for 30 min. Then, 150 µL of the solution from each well was transferred to assess the absorbance at 450 nm using a microplate reader (Multiskan GO, United States) to evaluate the cytotoxicity of the modified titanium. Additionally, at 1 day and 3 days, the growth and proliferation of cells on the modified titanium surface were examined using a live/dead staining kit (Keygen BioTECH). Live cells were stained with Calcein acetoxymethyl ester (Calcein AM), emitting green fluorescence, while dead cells were stained with propidium iodide (PI), emitting red fluorescence. The staining results were captured using an inverted fluorescence microscope (ECLIPSE Ti2, Nikon, China).

In 48-well plates, BMSCs or RAW264.7 cells (3,000 cells/well) were plated on the modified titanium. At 3 days, cell adhesion and morphology were evaluated through cytoskeletal staining. After fixing the cells with 4% paraformaldehyde for 10 min, they were washed with PBS. Cell membranes were disrupted using 0.1% Triton X-100 for 10 min. Subsequently, the cytoskeleton was stained with Rhodamine-Phalloidin (US EVERBRIGHT, Suzhou, China) at room temperature for 30 min. The nuclei were stained with 4′,6-diamino-2-phenylindole (DAPI, BioChannel Biological Technology Co. Ltd.). The modified titanium was transferred to the glass bottom culture plate (NEST Biotechnology, Wuxi, China), and then cytoskeleton was visualized using a fluorescence confocal microscopy (ZEISS LSM 980 with Airyscan 2, Jena, Germany).

### 2.5 Osteogenic effect of modified titanium

In 48-well plates, BMSCs (25,000 cells/well) were seeded on the modified titanium surfaces. After 24 h, once the cells fully adhered to the titanium, the culture medium was replaced with osteogenic induction medium. On day 7, the cells were fixed with 4% paraformaldehyde for 10 min and then stained using an ALP staining kit. On day 14, the cells were fixed with 4% paraformaldehyde for 10 min, and Sirius Red (G1472, Beijing Solarbio Science and Technology Co., Ltd.) staining was performed to assess collagen generation. After eluting collagen with a collagen elution solution (NaOH-0.2M: methanol = 1:1, 200µL/well), 150 µL of the solution was transferred from each well to measure the absorbance at 540 nm using a microplate reader (Multiskan GO, USA) for quantitative analysis. On day 21, the cells were fixed with 4% paraformaldehyde for 10 min and stained with Alizarin Red S (G1450, Beijing Solarbio Science and Technology Co., Ltd.) to evaluate calcium nodule formation. Following the elution of calcium nodules with a calcium nodule elution solution (10% cetylpyridinium chloride, 200µL/well), 150 µL of solution was collected from each well to measure the absorbance at 565 nm using the microplate reader for quantitative analysis.

In 6-well plates (Guangzhou Jet Bio-Filtration Co., Ltd.), BMSCs (25,000 cells/well) were seeded on the modified titanium. After 5 days of culture in the osteogenic induction medium, total RNA was extracted using the TRnaZol RNA Kit (New Cell & Molecular Biotech). The purity and concentration of the RNA were measured, and 1 µg of total RNA was used to synthesize cDNA with a reverse transcription kit (NovoScript^®^ miRNA First-Strand cDNA Synthesis and SYBR qPCR Kit, Novoprotein Scientific, Inc. Shanghai, China). Real-time PCR reactions were prepared using the SuperReal PreMix Plus (SYBR Green) kit and primers listed in Table 1. Amplification and detection were performed with a real-time fluorescent quantitative PCR instrument (LightCycler480, Roche), and the relative expression of osteogenic genes was analyzed using β-actin as the internal control.

**TABLE 1:** Primers sequences used for RT-PCR of BMSCs.

Gene	Forward primer sequences (5′-3′)	Reverse primer sequences (5′-3′)
OCN ALP	TTCTGCTCACTCTGCTGACC CCGGCTGGAGATGGACAAAT	GCCGGAGTCTGTTCACTACC TAGTCACAATGCCCACGGAC
COL-1	CCT​GGC​AAA​GAC​GGA​CTC​AA	CGG​CCA​CCA​TCT​TGA​GAC​TT
β-actin	GAC​CCA​GAT​CAT​GTT​TGA​GAC​CT	TCC​AGG​GAG​GAA​GAG​GAT​GC

*OCN*, osteocalcin; *ALP*, alkaline phosphatase; *COL-I*, collagen-1.

### 2.6 Immunomodulatory effect of modified titanium

In 6-well plates, RAW264.7 cells (1*10^ ^6^ cells/well) were seeded on the modified titanium surfaces. Lipopolysaccharide (LPS, final concentration of 1 µg/ml) was added to each group to simulate the inflammatory response stage. After 3 days, cells were collected, adjusted to a density of 1*10^ ^6^ cells/mL. Cells were washed twice with pre-cold DPBS. Cells were incubated with antibody to CCR7 (Hunan ProMab Biotechnologies Co., Ltd.) or antibody to CD206 for 30 min at room temperature in the dark, followed by washing and centrifugation. A secondary fluorescent antibody was added, and cells were incubated for another 30 min under the same conditions. A 1X Binding Buffer (400 µL/well) was added to each sample for analysis via flow cytometry (C6, BD).

In 6-well plates, RAW264.7 cells (10,000 cells/well) were seeded on the modified titanium. After 3 days, total RNA was extracted using the Trizol method. The purity and concentration of the RNA were measured, and 1 µg of total RNA was used to synthesize cDNA with the reverse transcription kit. Real-time PCR reactions were prepared using the SYBR Green kit and primers listed in Table 2. Amplification and detection were performed with a real-time fluorescent quantitative PCR instrument (LightCycler480, Roche), and the relative expression of polarization related genes was analyzed using β-actin as the internal control.

**TABLE 2:** Primers sequences used for RT-PCR of RAW264.7.

Gene	Forward primer sequences (5′-3′)	Reverse primer sequences (5′-3′)
TNF-α IL-1*β*	CCCACGTCGTAGCAAACCA GTGAAATGCCACCTTTTGACAGTGA	ACAAGGTACAACCCATCGGC CCACGGGAAAGACACAGGTAG
IL-6 IL-4	CTTCTTGGGACTGATGCTGGTGAC CATCGGCATTTTGAACGAG	TCTGTTGGGAGTGGTATCCTCTGTG ACGTTTGGCACATCCATCTC
IL-10 β-actin	AAGCTCCAAGACCAAGGTGTC GACCCAGATCATGTTTGAGACCT	TCGGAGAGAGGTACAAACGAG TCCAGGGAGGAAGAGGATGC

*TNF-α*, tumor necrosis factor α, *IL-1β*, interleukin-1*β*, *IL-6*, interleukin-6, *IL-4*, interleukin-4, *IL-10*, interleukin-10.

### 2.7 Subcutaneous implantation model and tissue staining

All animal experimentation was endorsed by the Animal Ethics Committee of Hunan Yaoda Testing Technology Co., Ltd. The protocols were performed following the guidelines of the Institutional Animal Use Committee of China. Totally 18 male Sprague-Dawley rats (SD rats, weighting 200–250 g) were randomly divided into three groups, which were implanted with three different materials: Ti, Ti@PTL, and Ti@PTL/Sr These groups were designated as the Ti group, Ti@PTL group, and Ti@PTL/Sr group, respectively. After anesthesia via intraperitoneal injection of 3% pentobarbital, a 1 cm longitudinal incision was made on the back of each rat. Subsequently, the materials were implanted between the skin and muscle layers. The rats were euthanized on day 7 and day 14 post-implantation, and the skin tissues surrounding the implants were excised and fixed in 4% paraformaldehyde.

The fixed skin tissues were sectioned and subjected to hematoxylin-eosin (H&E) staining, Masson staining, and immunohistochemical staining for CD31, iNOS, and CD163. These staining techniques were used to comprehensively assess the biocompatibility, pro-angiogenic, and immunomodulatory properties of the modified titanium *in vivo*.

### 2.8 Statistical analysis

The results were expressed as mean ± standard deviation, with each experimental group containing at least three parallel samples. One-way analysis of variance (ANOVA) was conducted to compare differences among the groups. All statistical analyses were performed using GraphPad Prism 9.5 (GraphPad Software, USA). Statistical significance levels were set at *p* < 0.05 (*p < 0.05, **p < 0.01, ***p < 0.001, ****p < 0.0001). Non-significant results were labeled as ns.

## 3 Results

### 3.1 Surface morphology of modified titanium

**FIGURE 1:**
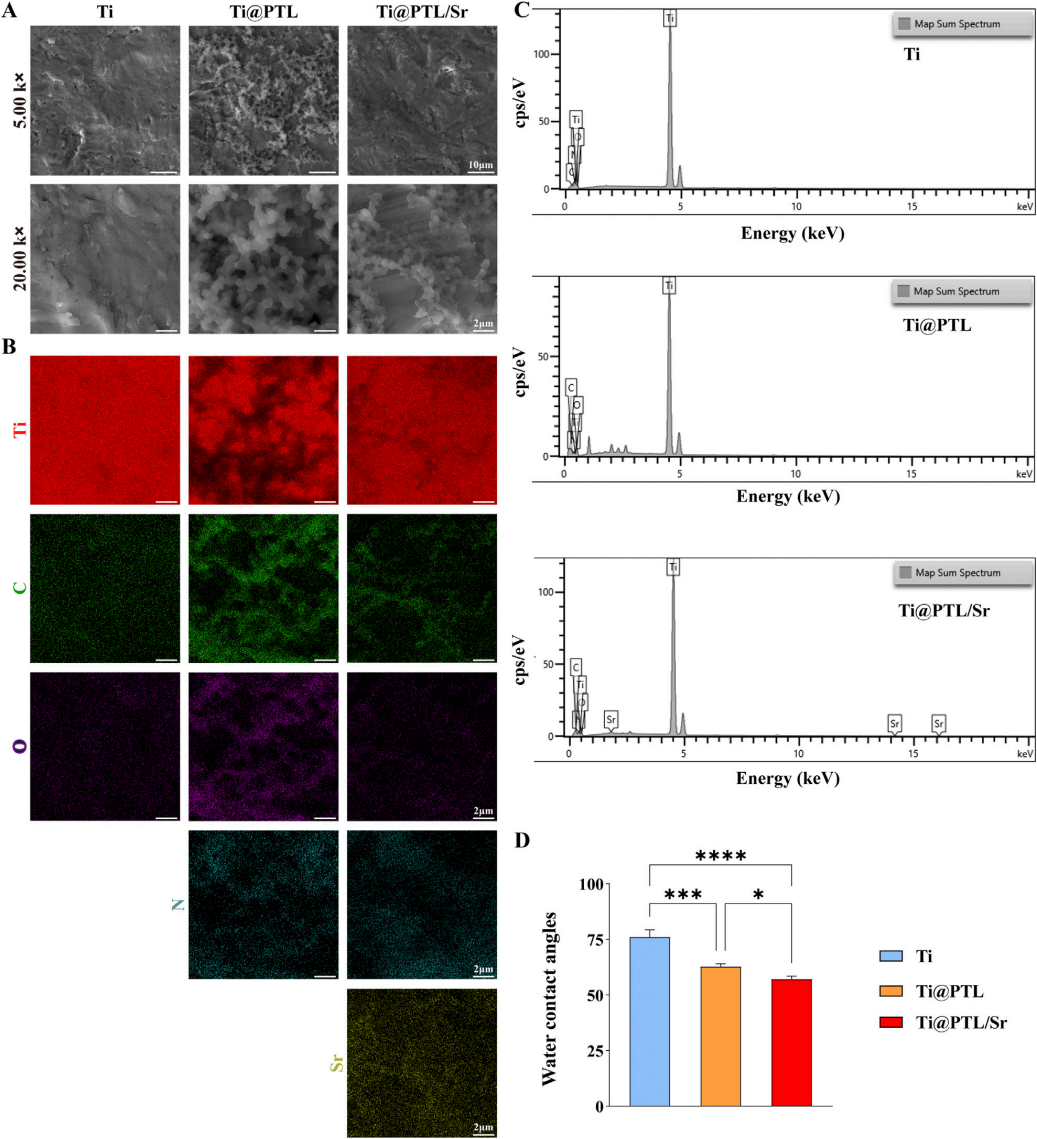
Surface Morphology and Element Distribution of Each Material. **(A)** Representative images of the surface morphology of each material. Magnification at 5.00k, scale bar = 10 µm. Magnification at 20.00 k, scale bar = 2 µm. **(B)** Representative images of the element distribution on each material surface. Scale bar = 2 µm. **(C)** Elemental analysis results. **(D)** Statistical results of water contact angle. (*p < 0.05, **p < 0.01, ***p < 0.001, ****p < 0.0001).

The surface morphology of each material was observed using scanning electron microscopy (SEM). The Ti surface showed no visible attachments, with unevenness likely due to incomplete polishing (Figure 1A). In contrast, the titanium surface coated with PTL exhibited a clear granular network structure (Figure 1A). At 20.00k magnification, the granules had an approximate diameter of 500 nm and appeared rough rather than perfectly spherical (Figure 1A).

After immersion in the SrCl_2_ solution, the granular network on Ti@PTL/Sr appeared less dense compared to Ti@PTL, likely due to the prolonged immersion and washing steps, which may have caused partial detachment of the coating (Figure 1A). Elemental distribution analyzed by EDS indicated stronger C, O, and N signals in Ti@PTL and Ti@PTL/Sr compared to Ti, corresponding to the PTL coating. The denser PTL coating in Ti@PTL produced stronger signals, as confirmed by the elemental analysis results (Figure 1C). In addition, Sr was detected on the Ti@PTL/Sr surface, although with relatively weak intensity (Figures 1B, C), suggesting that further validation of Sr^2^⁺ grafting would be necessary through XPS analysis (Section 3.2).

The hydrophilicity of the materials significantly affects cell adhesion and other cellular behaviors. Statistical results indicated that the water contact angle for Ti was 76.00° ± 3.27°, while the PTL-coated Ti had a reduced water contact angle of 62.79° ± 1.25°, suggesting that the PTL coating increased the hydrophilicity of Ti (Figure 1D). However, some studies have reported that PTL deposition may reduce hydrophilicity (He et al., 2023), indicating that the effect of the PTL coating on substrate hydrophilicity could be bidirectional and dependent on the inherent hydrophilicity of the substrate itself. The PTL coating grafted with Sr^2^⁺ further increased the hydrophilicity (57.09° ± 1.37°), demonstrating the potential for further functional modification of the PTL coating (Figure 1D).

### 3.2 Physicochemical properties of modified titanium

**FIGURE 2:**
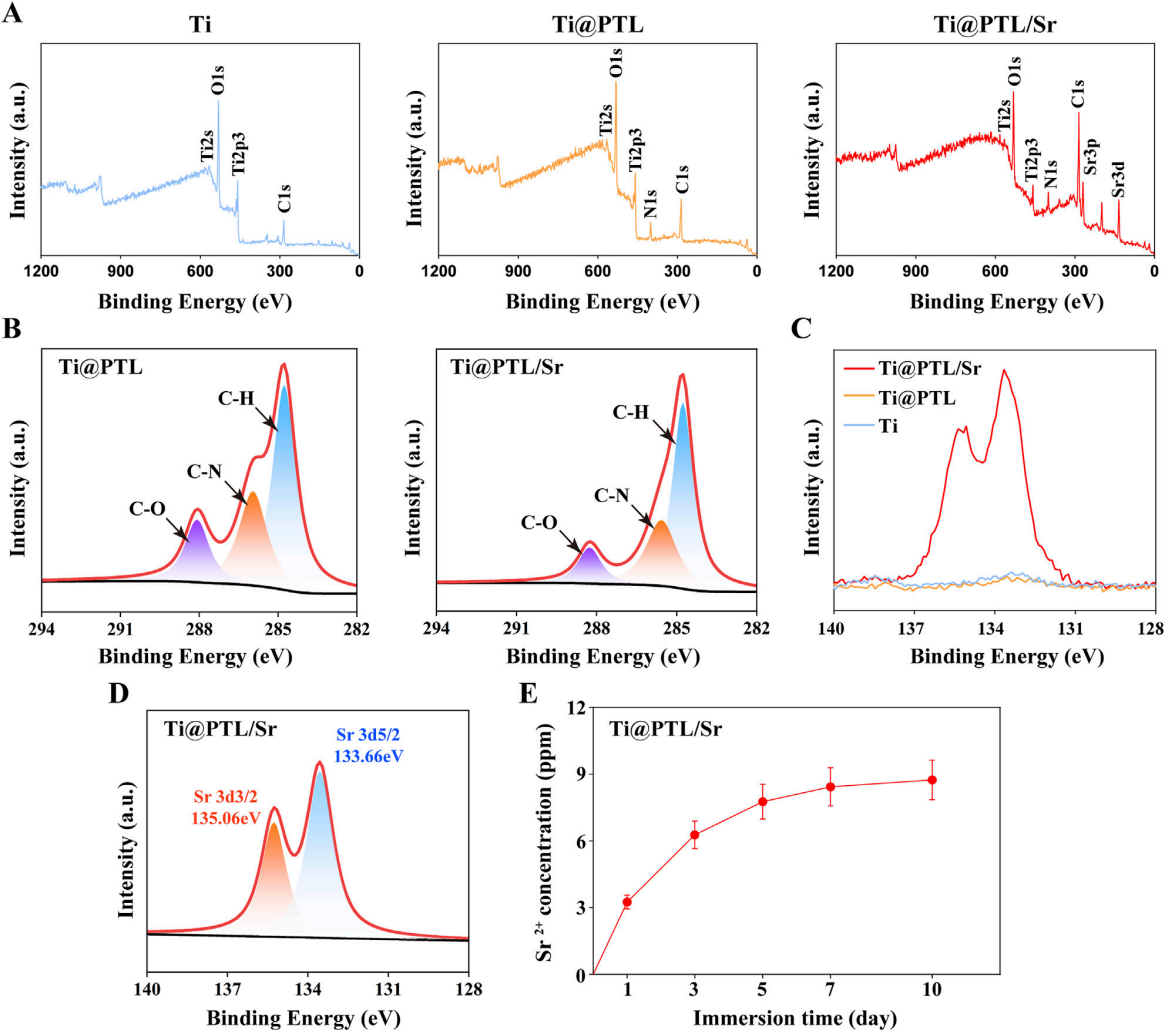
XPS Analysis Results and Sr^2+^ Release Profile of Modified Titanium. **(A)** XPS spectra of each material. **(B)** Peak fitting results of the C1s spectrum. **(C)** XPS spectra of each material in the 128–140 eV range. **(D)** Fitting results of the Sr3d peak on Ti@PTL/Sr. **(E)** Sr^2+^ release profile of Ti@PTL/Sr.

The X-ray photoelectron spectroscopy (XPS) results facilitated further investigation into the chemical composition of the modified titanium. XPS spectra for all groups showed the presence of Ti, C, and O elements (Figure 2A). The signal corresponding to the O element is likely due to the formation of titanium oxides on the surface, while the signal for C may stem from the detection environment. Compared to Ti, the additional peaks for N1s were observed on Ti@PTL and Ti@PTL/Sr (Figure 2A), which are associated with amino groups in lysozyme (He et al., 2020).

Furthermore, peak fitting of the C1s spectrum revealed three distinct peaks at 287.86 eV (C-O), 286.06 eV (C-N), and 284.76 eV (C-H) (Figure 2B), possibly attributed to aliphatic and aromatic hydrocarbon groups present on the PTL coating surface (Fang et al., 2021). The specific peak positions may be subject to deviations caused by background interference. The N1s and C1s peak spectral results confirm the successful adsorption of the PTL coating onto the Ti surface.

In the XPS spectra of Ti@PTL/Sr, additional Sr3d and Sr3p peaks were observed, corresponding to Sr^2^⁺ (Figure 2A). Analysis of the region between 128–140 eV revealed that only Ti@PTL/Sr showed clear peaks, and curve fitting identified Sr3d3/2 (135.06 eV) and Sr3d5/2 (133.66 eV) peaks, confirming the successful grafting of Sr^2^⁺ onto the PTL coating (Figures 2C, D). The PTL coating contains abundant carboxyl and hydroxyl groups, which facilitate the electrostatic adsorption of Sr^2^⁺ onto the surface (Yang et al., 2018).

Release study of Sr^2+^ from Ti@PTL/Sr demonstrated that over 60% of Sr^2+^ was completely released within the first 3 days, followed by a gradual release thereafter (Figure 2E). This indicates that the modified titanium can elicit corresponding bioactivity in the early stages by releasing sufficient amounts of Sr^2+^.

### 3.3 Modified titanium exhibits excellent biocompatibility *in vitro*

**FIGURE 3:**
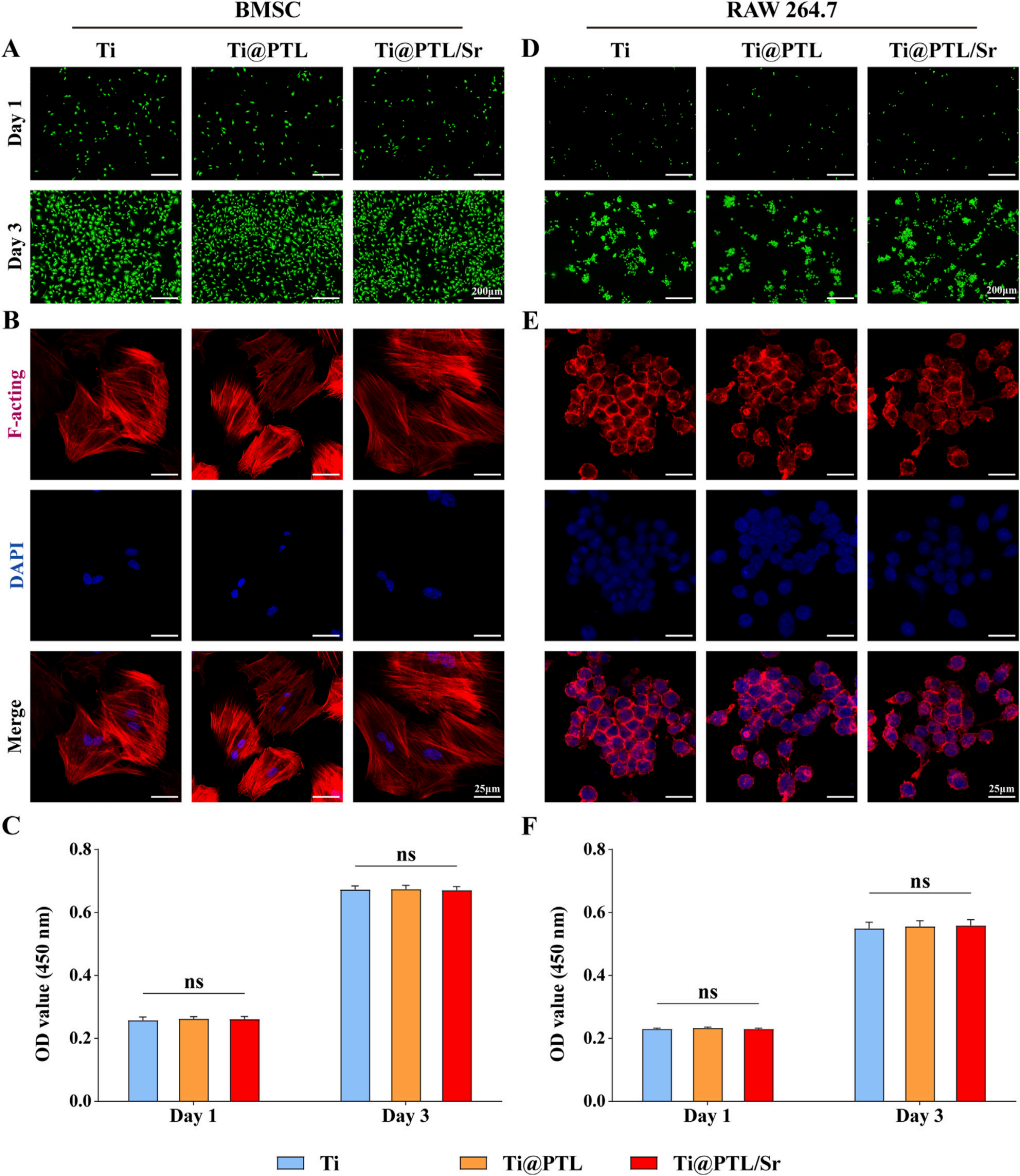
Growth of BMSCs and RAW264.7 Cells on Material Surfaces. **(A)** Live/dead staining results for BMSCs on material surfaces at day 1 and day 3. Representative images show live (green) and dead (red) BMSCs. Scale bar = 200 µm. **(B)** Morphology and adhesion of BMSCs on material surfaces. Representative images illustrate the cytoskeleton (red) and nucleus (blue). Scale bar = 25 µm. **(C)** CCK-8 assay results for BMSCs on material surfaces at day 1 and day 3. **(D)** Live/dead staining results for RAW264.7 cells on material surfaces at day 1 and day 3. Representative images show live (green) and dead (red) RAW264.7 cells. Scale bar = 200 µm. **(E)** Morphology and adhesion of RAW264.7 cells on material surfaces. Representative images illustrate the cytoskeleton (red) and nucleus (blue). Scale bar = 25 µm. **(F)** CCK-8 assay results for RAW264.7 cells on material surfaces at day 1 and day 3. (*p < 0.05, **p < 0.01, ***p < 0.001, ****p < 0.0001).

As orthopedic implants, possessing excellent biocompatibility is essential for their biological function. BMSCs and RAW264.7 cells were seeded on the material surfaces and cultured. Live/dead staining showed no significant cell death on day 1 or day 3 (Figures 3A, D). Normal cell proliferation was observed across all material surfaces. The absorbance results from the CCK-8 assay corroborated this observation, indicating that cell proliferation was not significantly affected by the different materials, and the culture results for both cell types were consistent (Figures 3C, F). This suggests that the presence of PTL and Sr^2+^ does not affect the biocompatibility of Ti, and all materials demonstrated good biocompatibility *in vitro*.

Furthermore, the CCK-8 assay results for the different materials were minimally different, maintaining similar levels on day 3. In BMSCs (Ti: 0.673 ± 0.011, Ti@PTL: 0.674 ± 0.012, Ti@PTL/Sr: 0.671 ± 0.012) and RAW264.7 cells (Ti: 0.549 ± 0.021, Ti@PTL: 0.556 ± 0.019, Ti@PTL/Sr: 0.558 ± 0.019), the trends were consistent, suggesting that the biological effects of Ti@PTL/Sr do not operate through pathways that influence cell proliferation (Figures 3C, F).

The surface properties of implants play a crucial role in cell adhesion, which influences cellular responses and the biological activity of the material. (Bai et al., 2021). The adhesion of osteoblasts to the surface of bone implants can promote early integration and repair of bone (Okuzu et al., 2017). Comparison of BMSC morphology on the three materials revealed more filopodia on Ti@PTL and Ti@PTL/Sr (Figure 3B). BMSCs spread widest on the Ti@PTL/Sr surface, indicating that the grafting of Sr^2+^ enhances the hydrophilicity of the coating and promotes the adhesion of BMSCs (Figure 3B).

RAW264.7 cells exhibited normal adhesion across all three materials and predominantly assumed a rounded shape, similar to M0 macrophages (Figure 3E). However, some cells displayed a spindle shape on Ti@PTL and Ti@PTL/Sr, resembling M2 macrophages (Figure 3E). Further experimental studies are needed to determine whether modified titanium influences the polarization of RAW264.7 cells (Section 3.5).

### 3.4 Modified titanium promotes osteogenic differentiation of BMSCs

**FIGURE 4:**
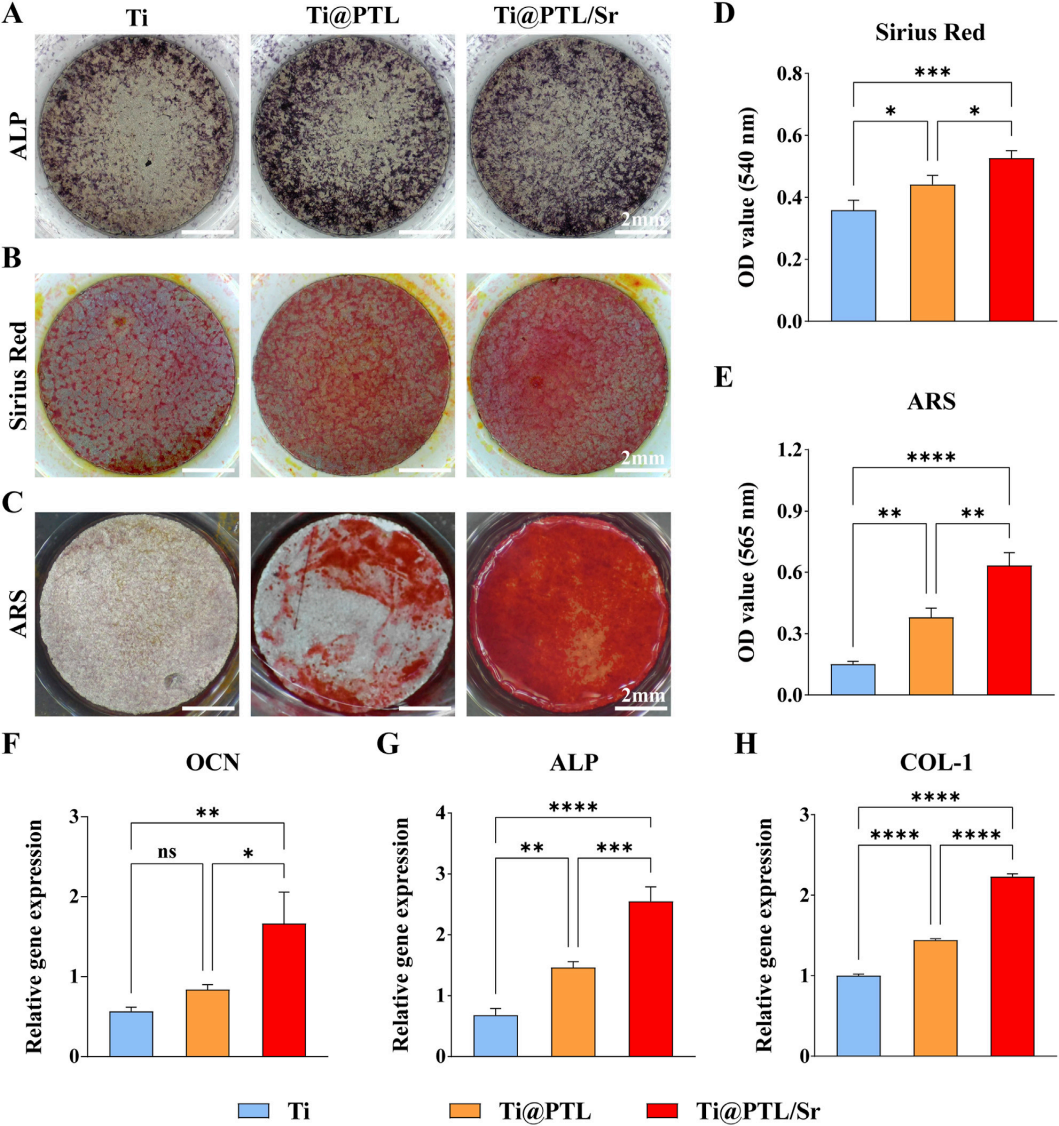
Osteogenic Differentiation of BMSCs on Material Surfaces. **(A)** Representative images of ALP staining results, where ALP exhibits a deep blue coloration after staining. Scale bar = 2 mm. **(B)** Representative images of Sirius Red staining results, where collagen appears orange-red after staining. Scale bar = 2 mm. **(C)** Representative images of Alizarin Red staining results, where mineralized nodules exhibit a red coloration after staining. Scale bar = 2 mm. **(D)** Quantitative analysis of Sirius Red staining results. **(E)** Quantitative analysis of Alizarin Red staining results. **(F)** Relative expression levels of the OCN gene. **(G)** Relative expression levels of the ALP gene. **(H)** Relative expression levels of the COL-1 gene. (*p < 0.05, **p < 0.01, ***p < 0.001, ****p < 0.0001).

Compared to Ti, both Ti@PTL and Ti@PTL/Sr exhibited deeper and more widespread coloration after staining with the ALP staining kit, indicating that BMSCs on Ti@PTL and Ti@PTL/Sr express the highest levels of ALP (Figure 4A). RT-PCR results confirmed that the expression of the ALP gene in both Ti@PTL and Ti@PTL/Sr groups was higher than that in the Ti group, with the highest expression observed in BMSCs on Ti@PTL/Sr (Figure 4G). Increased ALP expression promotes bone matrix mineralization and the osteogenesis process.

Sirius Red and Alizarin Red staining results demonstrated the superior osteogenic potential of Ti@PTL/Sr The formation of collagen and the deposition of mineralized nodules increased sequentially from Ti to Ti@PTL to Ti@PTL/Sr, with the most pronounced effects seen in Ti@PTL/Sr (Figures 4B, C). Quantitative analysis of Sirius Red staining revealed significant differences between Ti@PTL/Sr and Ti as well as Ti@PTL, and between Ti and Ti@PTL, suggesting that both the PTL coating and Sr^2+^ significantly promote collagen synthesis in BMSCs, with the latter being the predominant factor (Figure 4D). A similar trend was observed in the quantitative analysis of Alizarin Red staining, indicating that both the PTL coating and Sr^2+^ enhance calcium nodule formation in BMSCs, with Sr^2+^ being the primary factor (Figure 4E).

RT-PCR results demonstrated that Ti@PTL and Ti@PTL/Sr significantly upregulated the expression of the ALP and COL-1 genes, with Ti@PTL/Sr also significantly promoting the upregulation of the OCN gene (Figures 4F–H). The upregulation of the OCN gene facilitates the synthesis of osteocalcin, thereby aiding in calcium ion deposition (Yu et al., 2024). The upregulation of the COL-1 gene promotes the synthesis of type I collagen, ultimately leading to the formation of a dense collagen network (Zhang et al., 2024).

In summary, the PTL coating and Sr^2+^ can induce the osteogenic differentiation and bone formation of BMSCs by promoting the expression of osteogenesis-related genes (OCN, ALP, COL-1), with Sr^2+^ playing a dominant role.

### 3.5 Modified titanium regulates the polarization of RAW264.7 cells toward the M2 phenotype

Macrophages of the M0 type can differentiate into M1 and M2 phenotypes (Kazimierczak et al., 2021). Inflammatory macrophages (M1) secrete pro-inflammatory cytokines such as TNF-α, IL-1β, and IL-6. In contrast, M2 type macrophages secrete anti-inflammatory and repairing cytokines, including IL-4 and IL-10. Assessing the expression types and levels of these cytokines helps differentiate between M1 and M2 macrophages.

**FIGURE 5:**
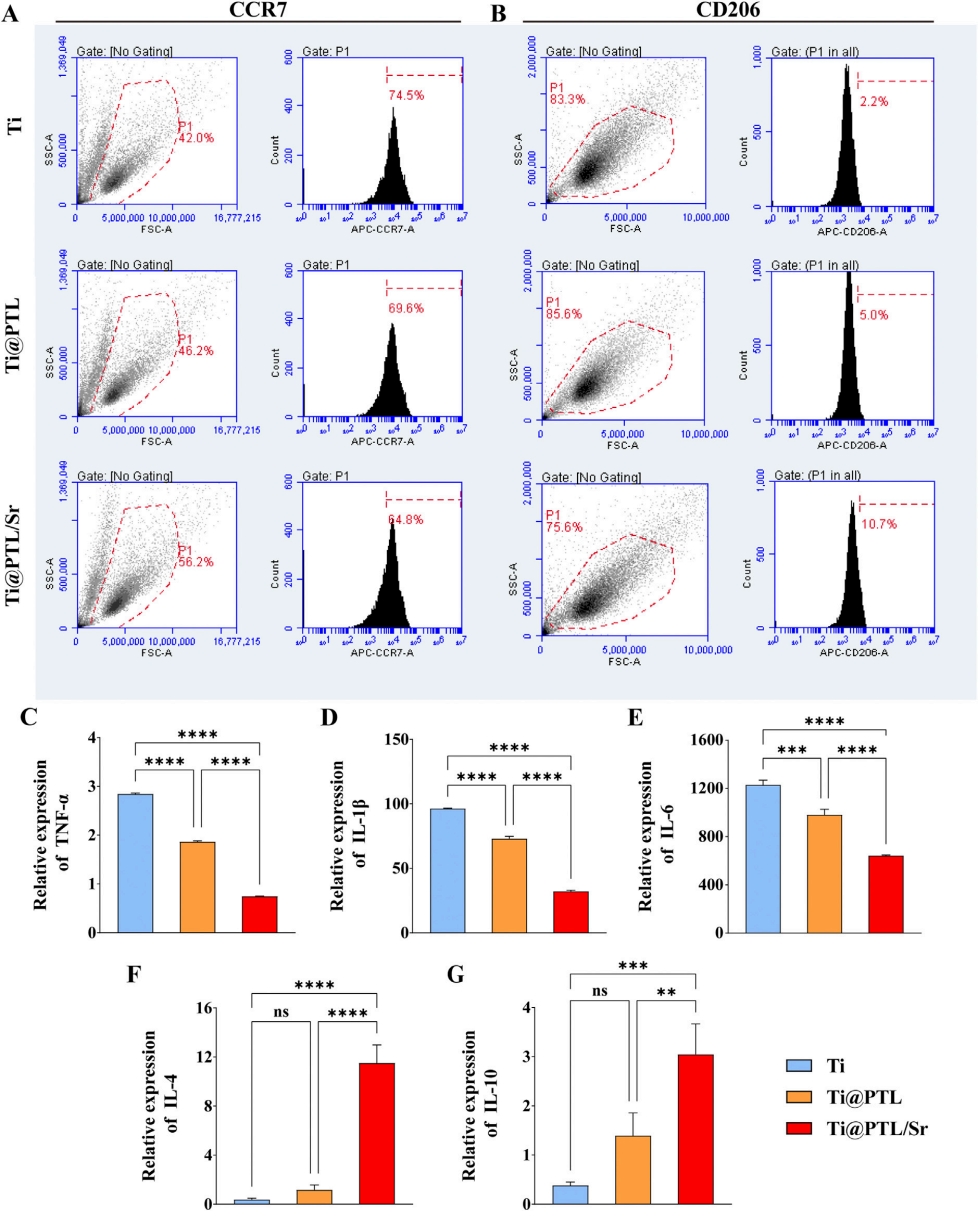
Polarization of RAW264.7 Cells on Material Surfaces. **(A)** Results of the CCR7-positive cell population, displayed as dot plots (left) and histograms (right). **(B)** Results of the CD206-positive cell population, presented as dot plots (left) and histograms (right). **(C–G)** Relative expression levels of TNF-α, IL-1β, IL-6, IL-4, and IL-10 genes, respectively. (*p < 0.05, **p < 0.01, ***p < 0.001, ****p < 0.0001).

Flow cytometry histograms reveal a sequential decrease in the proportion of CCR7-positive (M1 type) cells among the Ti, Ti@PTL, and Ti@PTL/Sr groups, with the most pronounced decrease observed in the Ti@PTL/Sr group (Figure 5A). Conversely, the proportion of CD206-positive (M2 type) cells increased progressively in the Ti, Ti@PTL, and Ti@PTL/Sr groups, showing the most significant increase in the Ti@PTL/Sr group (Figure 5B).

RT-PCR results indicate that both Ti@PTL and Ti@PTL/Sr significantly reduced the relative expression of TNF-α, IL-1β, and IL-6 genes compared to Ti, with the most notable effect seen in the Ti@PTL/Sr group (Figures 5C–E). Additionally, Ti@PTL/Sr significantly increased the relative expression of IL-4 and IL-10 genes compared to Ti and Ti@PTL (Figures 5F, G). Although the relative expression of IL-4 and IL-10 genes in Ti@PTL was higher than that in Ti, no statistically significant difference was observed between the two groups, suggesting that the PTL coating does not directly regulate the polarization of RAW264.7 cells to the M2 phenotype.

Overall, it is evident that Ti@PTL/Sr primarily promotes the differentiation of RAW264.7 cells to the M2 phenotype, with this process being largely mediated by Sr^2+^.

### 3.6 Modified titanium promotes tissue repair and angiogenesis *in vivo*

**FIGURE 6:**
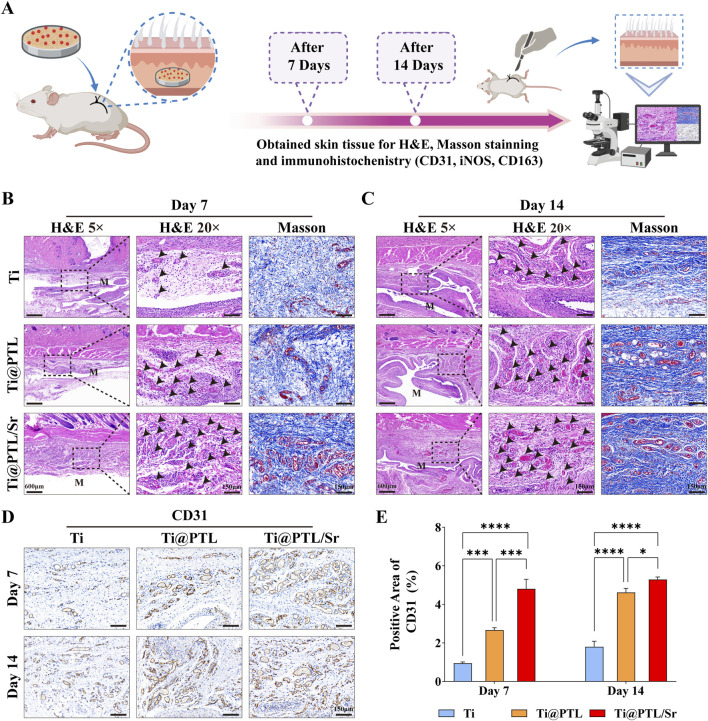
Assessment of Tissue Repair Effects of Each Material *in vivo* through Tissue Staining. **(A)** Preparation of subcutaneous implantation model and the tissue staining process. **(B)** Representative images of H&E staining on day 7 (Magnification at 5, left, scale bar = 600 μm. Magnification at 20, middle, scale bar = 150 μm) and Masson staining (Magnification at 20, right, scale bar = 150 μm). Black arrows indicate neovessels, and the M label denotes the approximate embedding position of the materials within the skin tissue. **(C)** Representative images of H&E staining on day 14 (Magnification at 5, left, scale bar = 600 μm. Magnification at 20, middle, scale bar = 150 μm) and Masson staining (Magnification at 20, right, scale bar = 150 μm). Black arrows again indicate neovessels, with the M label indicating the approximate embedding position of the materials in the skin tissue. **(D)** Representative images of CD31 immunohistochemistry, with positive result areas exhibiting a brown coloration. Scale bar = 150 μm. **(E)** Semi-quantitative analysis of CD31 immunohistochemistry results. (*p < 0.05, **p < 0.01, ***p < 0.001, ****p < 0.0001).

The preparation of subcutaneous implantation models and the tissue staining process are illustrated in Figure 6A. Comparing the H&E staining results of the Ti, Ti@PTL, and Ti@PTL/Sr groups, it is evident that the Ti group exhibited the most pronounced inflammatory hyperplasia on days 7 and 14, while the inflammatory hyperplasia in the Ti@PTL/Sr group maintained the lowest levels throughout the 14-day period (Figures 6B, C). Additionally, black arrows indicate that the Ti@PTL/Sr group exhibited the highest number of neovessels. Masson staining further demonstrated that the tissue repair effect surrounding the materials was most pronounced in the Ti@PTL/Sr group (Figures 6B, C). The results from both H&E and Masson staining indicate that the modified titanium Ti@PTL/Sr can promote tissue repair *in vivo*.

Angiogenesis enhances local circulation and establishes a favorable microenvironment for tissue repair (Ruan et al., 2023). CD31 is a marker of neovascularization. Immunohistochemical analysis of CD31 revealed that, on day 7, the Ti@PTL/Sr group exhibited elevated levels of CD31, showing a significant difference compared to both the Ti and Ti@PTL groups (Figures 6D, E). On day 14, CD31 levels in the Ti@PTL/Sr group remained high, while the Ti@PTL group also showed a notable increase in CD31 levels (Figures 6D, E). A comparison between the Ti@PTL and Ti@PTL/Sr groups indicated that the significant difference in CD31 levels primarily occurred on day 7, suggesting that Sr^2+^ plays a dominant role in significantly promoting CD31 expression during the early phase (days 3–5), followed by a contribution from the PTL coating to further enhance CD31 expression (Figures 6D, E). This finding aligns with the substantial release of Sr^2+^ during the initial 3–5 days (Figure 2E). The above results illustrate the excellent pro-angiogenic effects of the modified titanium Ti@PTL/Sr.

### 3.7 Modified titanium suppresses inflammation and regulates macrophage polarization toward the M2 phenotype *in vivo*

**FIGURE 7:**
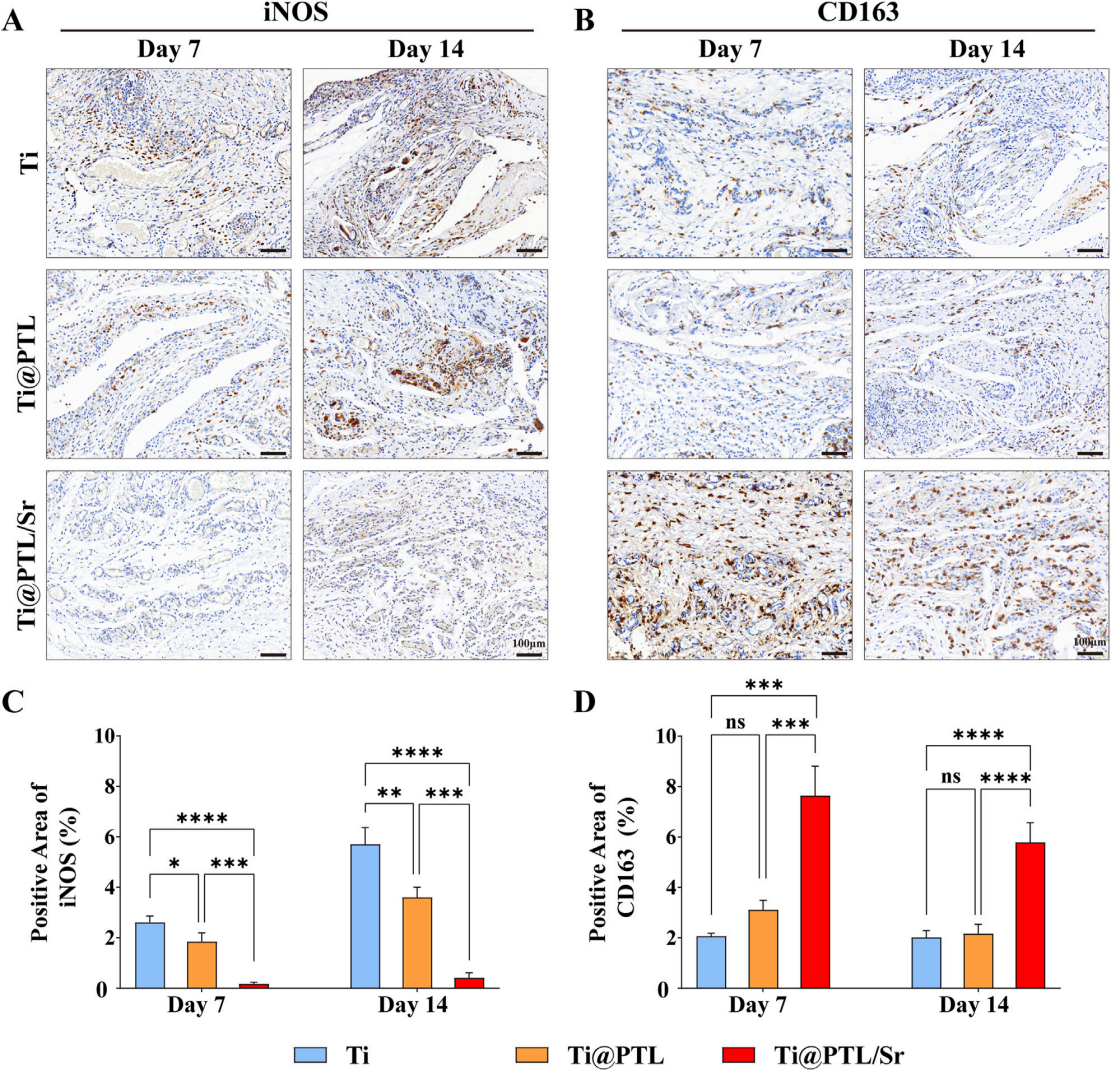
Evaluation of the Immunomodulatory Effects of Various Materials *in vivo* through Immunohistochemical Staining. **(A)** Representative images of iNOS immunohistochemical staining, with positive result areas exhibiting a brown coloration, indicating M1 macrophages. Scale bar = 100 μm. **(B)** Representative images of CD163 immunohistochemical staining, with positive result areas also exhibiting a brown coloration, indicating M2 macrophages. Scale bar = 100 μm. **(C)** Semi-quantitative analysis of iNOS immunohistochemical results. **(D)** Semi-quantitative analysis of CD163 immunohistochemical results. (*p < 0.05, **p < 0.01, ***p < 0.001, ****p < 0.0001).

Inducible nitric oxide synthase (iNOS) is one of the markers of M1 macrophages, while CD163 is a marker of M2 macrophages (Takabatake et al., 2024). A comparison of the immunohistochemical results for iNOS on days 7 and 14 revealed that the expression levels of iNOS in both the Ti and Ti@PTL groups increased over time (Figures 7A, C). The recruitment of M1 macrophages in these groups led to exacerbated local inflammation, which correlates with increased inflammatory hyperplasia (Figures 6B, C). Conversely, the immunohistochemical results for CD163 indicated that CD163 levels remained low in both the Ti and Ti@PTL groups on days 7 and 14, further suggesting that the PTL coating does not directly promote the polarization of macrophages towards the M2 phenotype (Figures 7B, D). In the Ti@PTL/Sr group, iNOS expression levels remained low on both days 7 and 14, indicating a reduction in inflammatory responses mediated by Sr^2+^ (Figures 7A, C). The Ti@PTL/Sr group exhibited the highest level of CD163 expression on day 7, which declined by day 14 (Figures 7B, D). This fluctuation in CD163 expression suggests that Sr^2+^ may exert its immunomodulatory effects by early inhibition of the inflammatory process rather than through a sustained effect. The combination of high CD163 expression and low iNOS expression in the Ti@PTL/Sr group indicates that the modified titanium Ti@PTL/Sr can regulate macrophage polarization towards the M2 phenotype *in vivo*, thereby exerting immunomodulatory effects.

## 4 Discussion

Designing high-performance orthopedic implant materials to replace autologous bone grafts for the repair of bone defects presents a significant challenge. Implant performance is increasingly studied beyond osteogenesis, angiogenesis, and antibacterial effects, with a growing focus on bone immunomodulation in the early stages post-implantation. This study aimed to improve the bioactivity of Ti by depositing a PTL coating to graft Sr^2+^, thereby creating a modified titanium. The expectations are that the modified titanium will exhibit enhanced osteogenic properties and immunoregulatory functions, contributing effectively to bone repair. Characterization results confirmed the successful deposition of the PTL coating and the effective grafting of Sr^2+^, indicating the feasibility of the proposed construction scheme for modified titanium.

Poor implant-bone integration is one of the key complications of orthopedic implants. Surface modifications that accelerate early osseointegration between the implant and bone are critical to improving the success rates of implants (Wei et al., 2020). The surface morphology and roughness of materials can influence the degree of osseointegration, as effective contact between the implant and bone tissue can reduce the likelihood of implant loosening and promote osteogenesis (Heo et al., 2016; Okuzu et al., 2017). Contact osteogenesis involves the recruitment and migration of osteoblasts and the subsequent bone formation by these cells, leading to bone apposition on the implant surface and ensuring optimal bone integration (Jin et al., 2019). The aliphatic and aromatic groups present on the PTL coating have been shown to improve the hydrophobicity of highly hydrophilic materials. In contrast, the hydrophobicity of PTL-coated Ti surfaces is reduced compared to uncoated Ti (Figure 1D). The grafting of hydrophilic polymers on the PTL coating can result in superhydrophilicity (Gao et al., 2016). Utilizing the PTL coating as a grafting surface allows for further modifications, even leading to the formation of hydroxyapatite coatings in simulated body fluids, suggesting that the PTL coating can facilitate the integration between the implant and bone (Yang et al., 2018; Lan et al., 2024). In this study, the PTL coating enhanced hydrophilicity after the grafting of Sr^2+^, which promoted the adhesion of BMSCs on the Ti@PTL/Sr surface (Figure 1D). Additionally, the roughened surface provided by the PTL coating favors the integration of titanium with bone tissue, potentially reducing the likelihood of loosening of the modified titanium.

In this study, the PTL coating and Sr^2+^ had minimal effects on cell proliferation. Live/dead staining and CCK-8 assay results indicated that the proliferation of BMSCs and RAW264.7 cells on Ti, Ti@PTL, and Ti@PTL/Sr materials was nearly at the same level (Figures 3A, C, D, F). Some findings supported this result (Zhou et al., 2018).However, there were also studies indicating a proliferative effect of Sr^2+^ (Huang et al., 2024; Miao et al., 2024). The conflicting results could stem from different material preparation processes and the actual concentrations and release rates of Sr^2+^ from the materials. This underscores the need for further research to refine the concentration of Sr^2+^ during material preparation.

Sr^2+^ enhances the expression of osteogenesis-related genes such as ALP, OCN, and BSP by activating the mitogen-activated protein kinase (MAPK) signaling pathway and promoting the phosphorylation of ERK1/2, thus facilitating osteoblast proliferation, matrix mineralization, and calcified nodule formation (Aimaiti et al., 2020; Sun et al., 2021). However, the effects of Sr^2+^ on osteoblast proliferation and differentiation are dose-dependent (Bussola Tovani et al., 2023). RT-PCR results indicated that, at the release concentration of Sr^2+^ in this study, it significantly promoted the expression of osteogenesis-related genes (OCN, ALP, COL-1), thereby inducing osteogenic differentiation and bone formation of BMSCs (Figures 4F, G). The effects of different concentrations of Sr^2+^ on the expression of osteogenesis-related genes warrant further investigation, and considering clinical applicability is also necessary (Aimaiti et al., 2017).

Both *in vitro* cell experiments and subcutaneous implantation model results indicated that modified titanium Ti@PTL/Sr promotes the polarization of macrophages towards the M2 phenotype. RT-PCR results showed that Ti@PTL/Sr significantly inhibited the expression of inflammatory factor-related genes (TNF-α, IL-1β, IL-6) in macrophages while significantly promoting the expression of anti-inflammatory factor-related genes (IL-4, IL-10) in macrophages (Figures 5C–G). Furthermore, the results indicated that the PTL coating does not directly promote the polarization of macrophages towards the M2 phenotype, suggesting that Sr^2+^ plays a dominant role in this process. Results has supported that Sr^2+^ modulates the immune state by regulating the expression of various cytokines in macrophages. Due to limitations in experimental conditions, the RT-PCR experiments only selected representative genes, which do not fully elucidate the immunomodulatory effects of Sr^2+^.

Additionally, H&E staining and CD31 immunohistochemistry results demonstrated that modified titanium Ti@PTL/Sr effectively promotes neovascularization (Figures 6B–E). Reports indicate that Sr^2+^ released from biomaterials can enhance the expression of pro-angiogenic factors (Liu et al., 2011). However, some experiments suggest that Sr^2+^ does not directly promote angiogenesis but rather facilitates early vascularization by inducing M2 macrophage activity at the implantation site (Miao et al., 2023). In the subcutaneous implantation model, Ti@PTL/Sr exhibited high levels of CD31 and CD163 expression, making it challenging to accurately attribute the pro-angiogenic effects directly to Sr^2+^ or to M2 macrophages. Therefore, further investigation is needed to delineate the mechanisms by which Sr^2+^ promotes angiogenesis. While Ti@PTL demonstrated favorable angiogenic effects on day 14, the underlying mechanisms of the PTL coating’s pro-angiogenic effects remain unclear and require further study (Figures 6D, E). Overall, the Ti@PTL/Sr modifications primarily facilitate neovascularization through the release of Sr^2+^ in the early phase, with subsequent angiogenesis mediated by the PTL coating.

To confirm the osteogenic effects of modified titanium Ti@PTL/Sr *in vivo*, it is necessary to conduct animal experiments using modified titanium as orthopedic implant materials for bone defect repair, which will be the focus of future research.

## 5 Conclusion

In this study, we deposited phase-transited lysozyme (PTL) onto the surface of titanium (Ti) to construct a functional coating, successfully grafting Sr^2+^ from SrCl_2_ solution onto this coating, resulting in multifunctional modified titanium Ti@PTL/Sr Characterization results confirmed the successful adsorption of the PTL coating and the successful grafting of Sr^2+^ onto the modified titanium Ti@PTL/Sr *In vitro* cellular experiments demonstrated that the modified titanium exhibited excellent biocompatibility and promoted the polarization of M2 macrophages, as well as the osteogenic differentiation of BMSCs. Subcutaneous implantation model *in vivo* further validated the favorable immunomodulatory and angiogenic properties of the modified titanium, contributing positively to tissue repair. In summary, modified titanium Ti@PTL/Sr can exert immunomodulatory effects and promote bone defect repair, showing promise as an orthopedic implant material. Further mechanistic exploration and studies using animal models will enhance the understanding of the clinical applicability of modified titanium, which will be the focus of future research.

## Data Availability

The original contributions presented in the study are included in the article/supplementary material, further inquiries can be directed to the corresponding authors.
